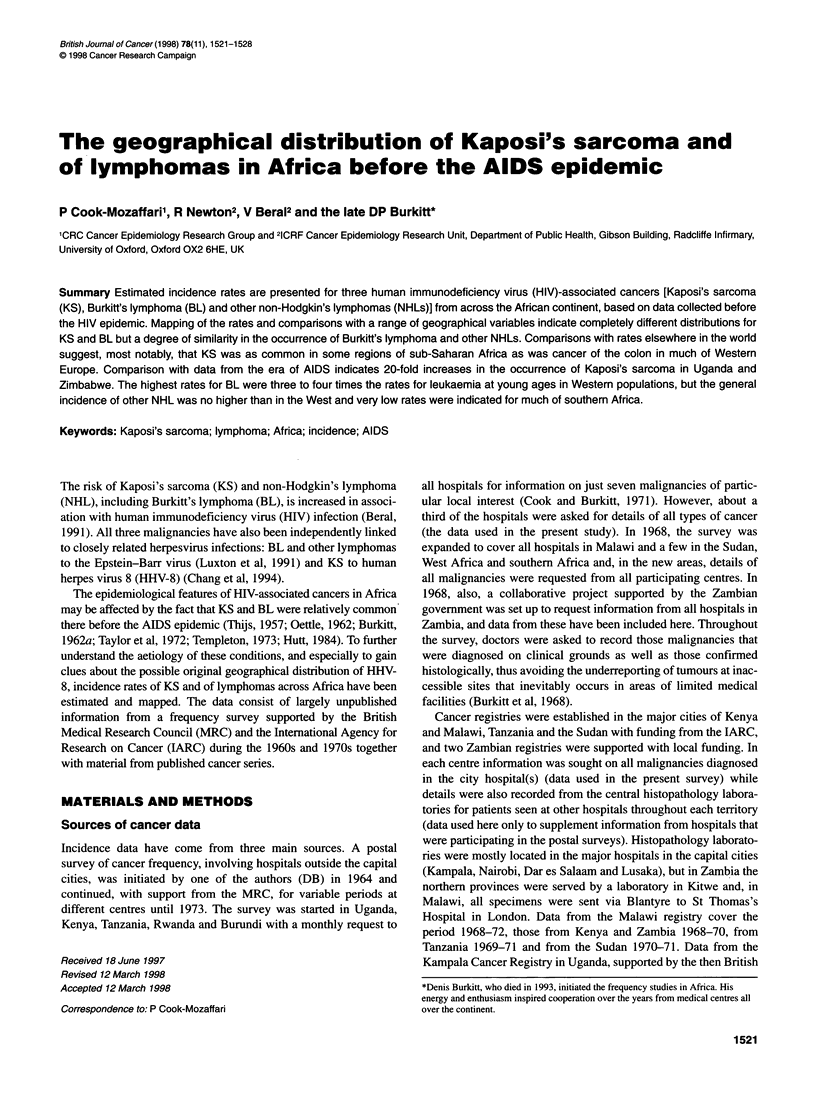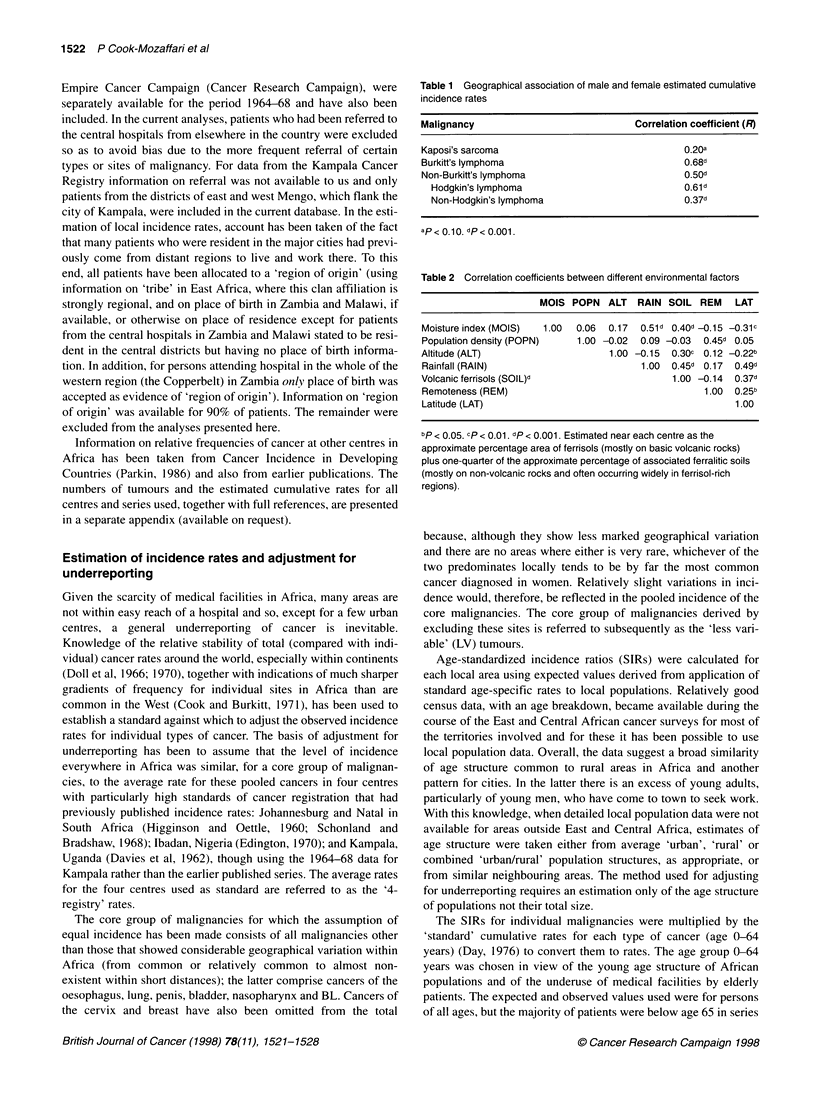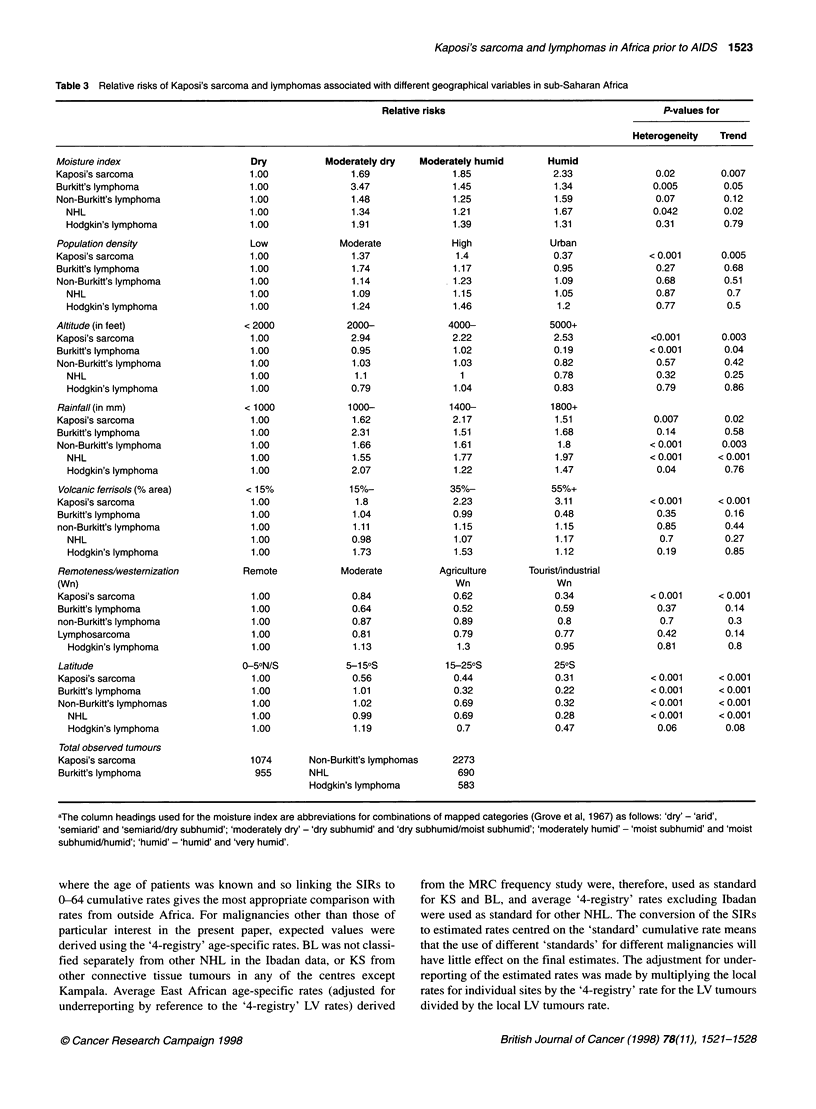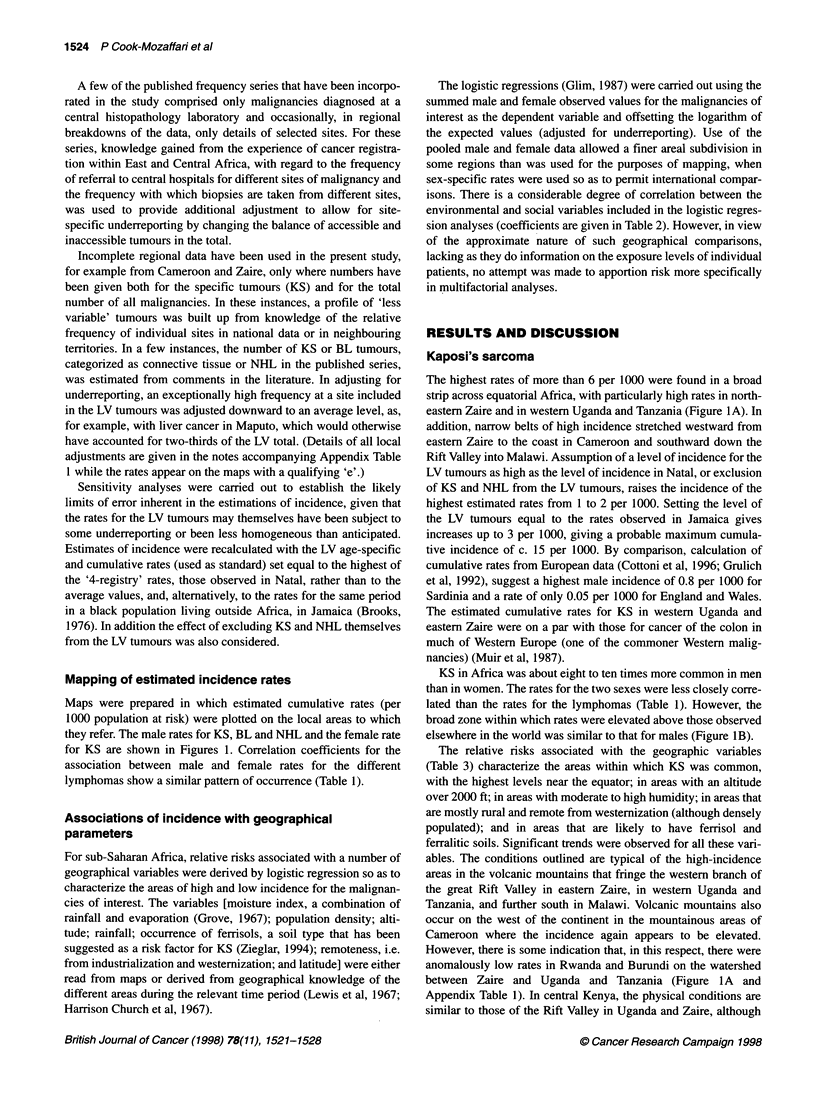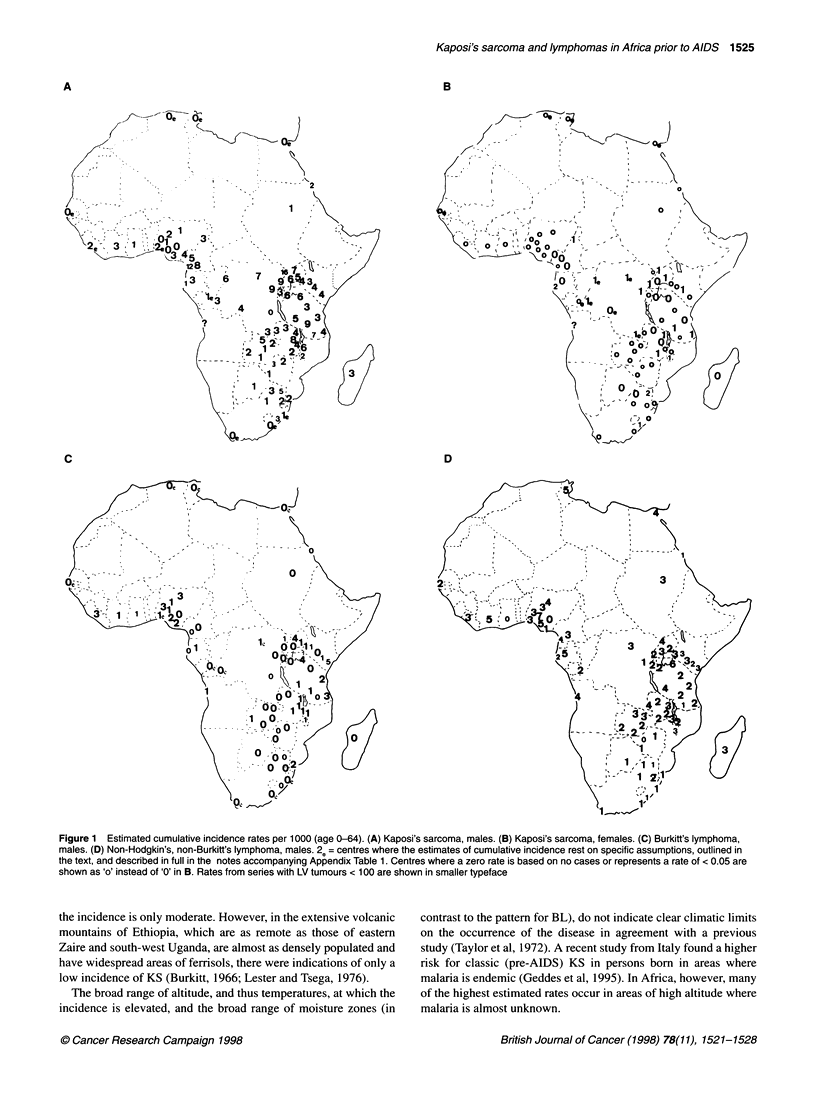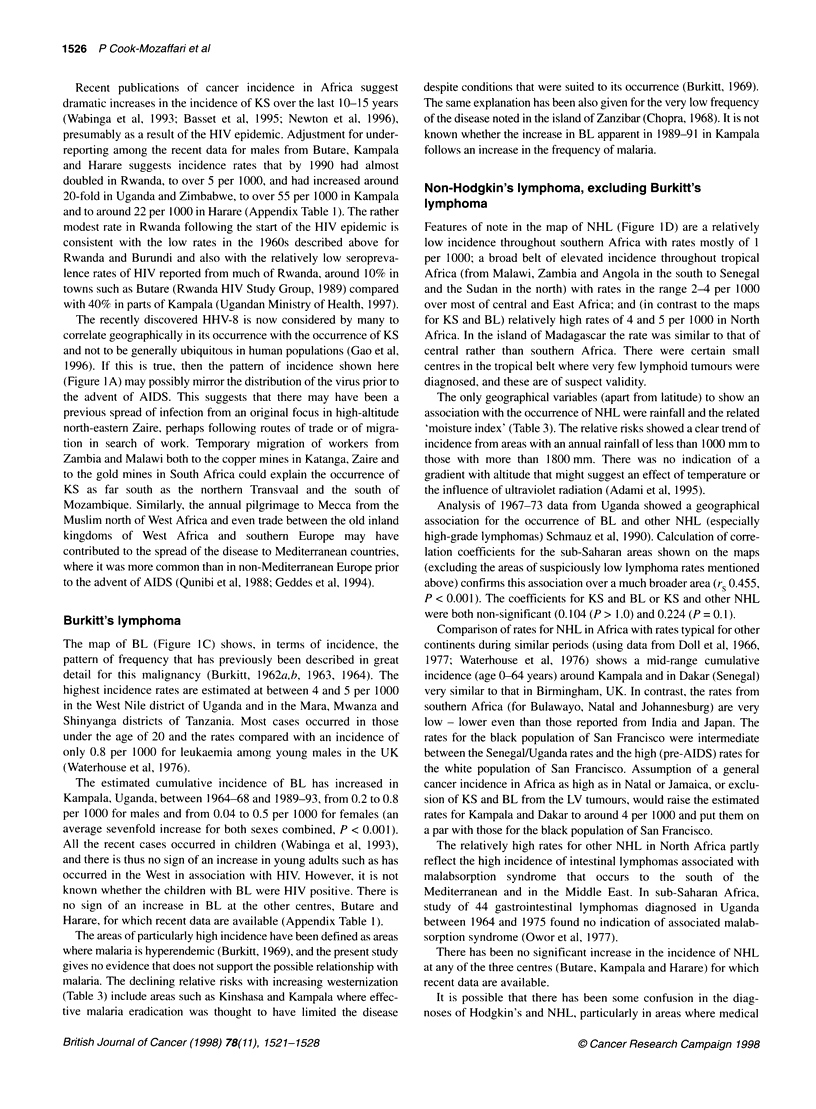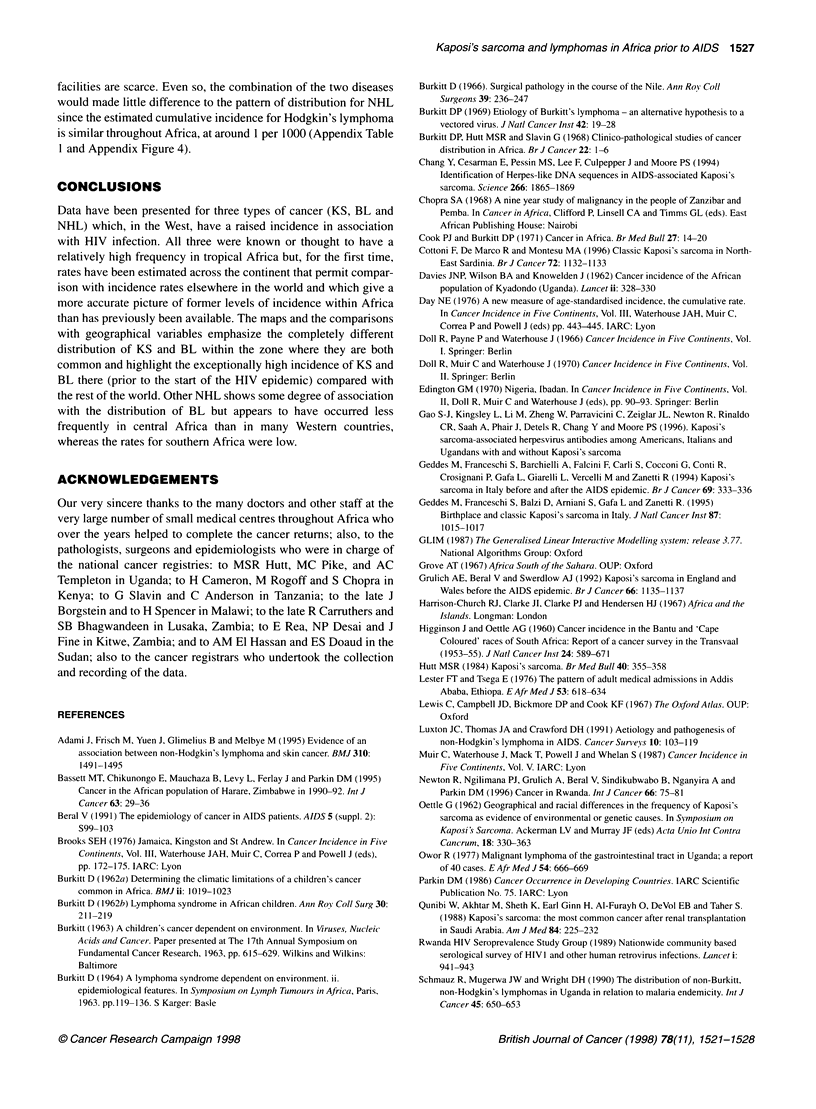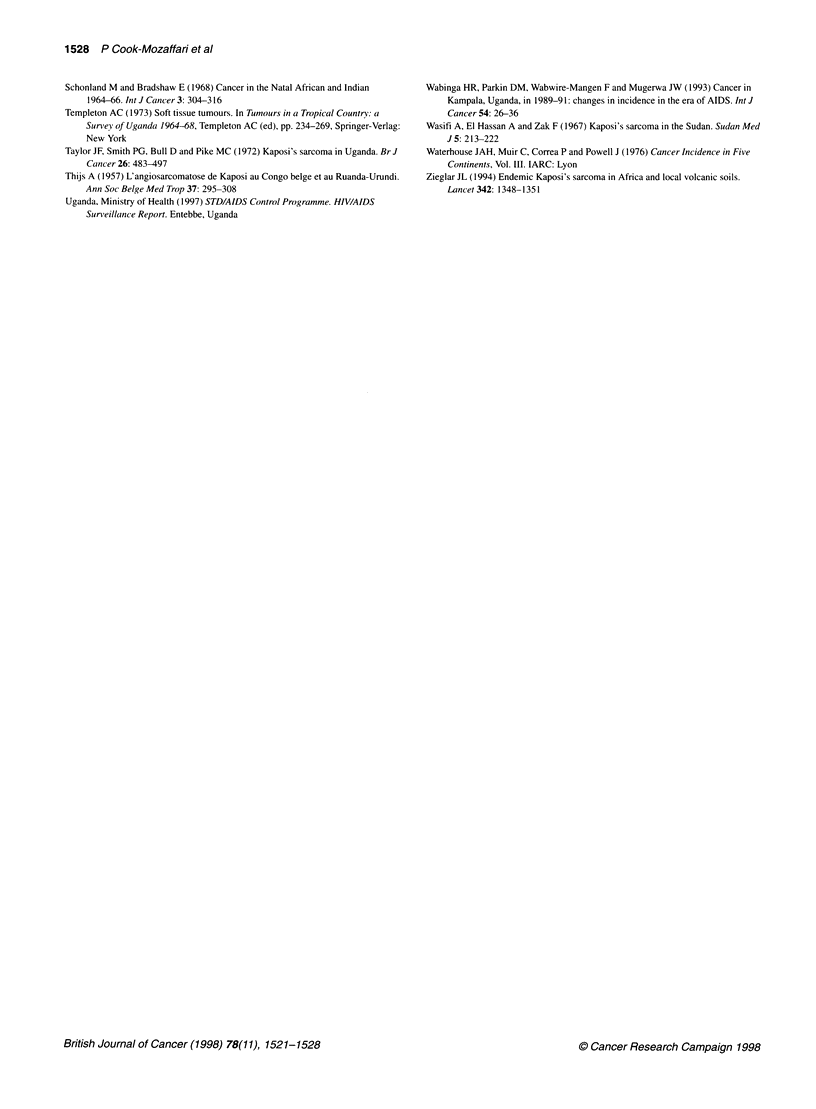# The geographical distribution of Kaposi's sarcoma and of lymphomas in Africa before the AIDS epidemic.

**DOI:** 10.1038/bjc.1998.717

**Published:** 1998-12

**Authors:** P. Cook-Mozaffari, R. Newton, V. Beral, D. P. Burkitt

**Affiliations:** CRC Cancer Epidemiology Research Group, Department of Public Health, Radcliffe Infirmary, University of Oxford, UK.

## Abstract

Estimated incidence rates are presented for three human immunodeficiency virus (HIV)-associated cancers [Kaposi's sarcoma (KS), Burkitt's lymphoma (BL) and other non-Hodgkin's lymphomas (NHLs)] from across the African continent, based on data collected before the HIV epidemic. Mapping of the rates and comparisons with a range of geographical variables indicate completely different distributions for KS and BL but a degree of similarity in the occurrence of Burkitt's lymphoma and other NHLs. Comparisons with rates elsewhere in the world suggest, most notably, that KS was as common in some regions of sub-Saharan Africa as was cancer of the colon in much of Western Europe. Comparison with data from the era of AIDS indicates 20-fold increases in the occurrence of Kaposi's sarcoma in Uganda and Zimbabwe. The highest rates for BL were three to four times the rates for leukaemia at young ages in Western populations, but the general incidence of other NHL was no higher than in the West and very low rates were indicated for much of southern Africa.


British Journal of Cancer (1998) 78(11), 1521–1528
© 1998 Cancer Research Campaign

### The geographical distribution of Kaposi's sarcoma and of lymphomas in Africa before the AIDS epidemic

**P Cook-Mozaffari[1], R Newton[2], V Beral[2] and the late DP Burkitt***

[1]CRC Cancer Epidemiology Research Group and [2]ICRF Cancer Epidemiology Research Unit, Department of Public Health, Gibson Building, Radcliffe Infirmary, University of Oxford, Oxford OX2 6HE, UK

**Summary** Estimated incidence rates are presented for three human immunodeficiency virus (HIV)-associated cancers [Kaposi's sarcoma (KS), Burkitt's lymphoma (BL) and other non-Hodgkin's lymphomas (NHLs)] from across the African continent, based on data collected before the HIV epidemic. Mapping of the rates and comparisons with a range of geographical variables indicate completely different distributions for KS and BL but a degree of similarity in the occurrence of Burkitt's lymphoma and other NHLs. Comparisons with rates elsewhere in the world suggest, most notably, that KS was as common in some regions of sub-Saharan Africa as was cancer of the colon in much of Western Europe. Comparison with data from the era of AIDS indicates 20-fold increases in the occurrence of Kaposi's sarcoma in Uganda and Zimbabwe. The highest rates for BL were three to four times the rates for leukaemia at young ages in Western populations, but the general incidence of other NHL was no higher than in the West and very low rates were indicated for much of southern Africa.

**Keywords:** Kaposi's sarcoma; lymphoma; Africa; incidence; AIDS

The risk of Kaposi's sarcoma (KS) and non-Hodgkin's lymphoma (NHL), including Burkitt's lymphoma (BL), is increased in association with human immunodeficiency virus (HIV) infection (Beral, 1991). All three malignancies have also been independently linked to closely related herpesvirus infections: BL and other lymphomas to the Epstein–Barr virus (Luxton et al, 1991) and KS to human herpes virus 8 (HHV-8) (Chang et al, 1994).

The epidemiological features of HIV-associated cancers in Africa may be affected by the fact that KS and BL were relatively common there before the AIDS epidemic (Thijs, 1957; Oettle, 1962; Burkitt, 1962*a*; Taylor et al, 1972; Templeton, 1973; Hutt, 1984). To further understand the aetiology of these conditions, and especially to gain clues about the possible original geographical distribution of HHV-8, incidence rates of KS and of lymphomas across Africa have been estimated and mapped. The data consist of largely unpublished information from a frequency survey supported by the British Medical Research Council (MRC) and the International Agency for Research on Cancer (IARC) during the 1960s and 1970s together with material from published cancer series.

### MATERIALS AND METHODS

### Sources of cancer data

Incidence data have come from three main sources. A postal survey of cancer frequency, involving hospitals outside the capital cities, was initiated by one of the authors (DB) in 1964 and continued, with support from the MRC, for variable periods at different centres until 1973. The survey was started in Uganda, Kenya, Tanzania, Rwanda and Burundi with a monthly request to all hospitals for information on just seven malignancies of particular local interest (Cook and Burkitt, 1971). However, about a third of the hospitals were asked for details of all types of cancer (the data used in the present study). In 1968, the survey was expanded to cover all hospitals in Malawi and a few in the Sudan, West Africa and southern Africa and, in the new areas, details of all malignancies were requested from all participating centres. In 1968, also, a collaborative project supported by the Zambian government was set up to request information from all hospitals in Zambia, and data from these have been included here. Throughout the survey, doctors were asked to record those malignancies that were diagnosed on clinical grounds as well as those confirmed histologically, thus avoiding the underreporting of tumours at inaccessible sites that inevitably occurs in areas of limited medical facilities (Burkitt et al, 1968).

Cancer registries were established in the major cities of Kenya and Malawi, Tanzania and the Sudan with funding from the IARC, and two Zambian registries were supported with local funding. In each centre information was sought on all malignancies diagnosed in the city hospital(s) (data used in the present survey) while details were also recorded from the central histopathology laboratories for patients seen at other hospitals throughout each territory (data used here only to supplement information from hospitals that were participating in the postal surveys). Histopathology laboratories were mostly located in the major hospitals in the capital cities (Kampala, Nairobi, Dar es Salaam and Lusaka), but in Zambia the northern provinces were served by a laboratory in Kitwe and, in Malawi, all specimens were sent via Blantyre to St Thomas's Hospital in London. Data from the Malawi registry cover the period 1968–72, those from Kenya and Zambia 1968–70, from Tanzania 1969–71 and from the Sudan 1970–71. Data from the Kampala Cancer Registry in Uganda, supported by the then British

*Received 18 June 1997*
*Revised 12 March 1998*
*Accepted 12 March 1998*

*Correspondence to:* P Cook-Mozaffari

*Denis Burkitt, who died in 1993, initiated the frequency studies in Africa. His energy and enthusiasm inspired cooperation over the years from medical centres all over the continent.

Empire Cancer Campaign (Cancer Research Campaign), were separately available for the period 1964–68 and have also been included. In the current analyses, patients who had been referred to the central hospitals from elsewhere in the country were excluded so as to avoid bias due to the more frequent referral of certain types or sites of malignancy. For data from the Kampala Cancer Registry information on referral was not available to us and only patients from the districts of east and west Mengo, which flank the city of Kampala, were included in the current database. In the estimation of local incidence rates, account has been taken of the fact that many patients who were resident in the major cities had previously come from distant regions to live and work there. To this end, all patients have been allocated to a 'region of origin' (using information on 'tribe' in East Africa, where this clan affiliation is strongly regional, and on place of birth in Zambia and Malawi, if available, or otherwise on place of residence except for patients from the central hospitals in Zambia and Malawi stated to be resident in the central districts but having no place of birth information. In addition, for persons attending hospital in the whole of the western region (the Copperbelt) in Zambia *only* place of birth was accepted as evidence of 'region of origin'). Information on 'region of origin' was available for 90% of patients. The remainder were excluded from the analyses presented here.

Information on relative frequencies of cancer at other centres in Africa has been taken from Cancer Incidence in Developing Countries (Parkin, 1986) and also from earlier publications. The numbers of tumours and the estimated cumulative rates for all centres and series used, together with full references, are presented in a separate appendix (available on request).

**Table 1** Geographical association of male and female estimated cumulative incidence rates

| Malignancy | Correlation coefficient ($R$) |
|---|---|
| Kaposi's sarcoma | 0.20[a] |
| Burkitt's lymphoma | 0.68[d] |
| Non-Burkitt's lymphoma | 0.50[d] |
| Hodgkin's lymphoma | 0.61[d] |
| Non-Hodgkin's lymphoma | 0.37[d] |

[a]$P < 0.10$. [d]$P < 0.001$.

**Table 2** Correlation coefficients between different environmental factors

| | MOIS | POPN | ALT | RAIN | SOIL | REM | LAT |
|---|---|---|---|---|---|---|---|
| Moisture index (MOIS) | 1.00 | 0.06 | 0.17 | 0.51[d] | 0.40[d] | –0.15 | –0.31[c] |
| Population density (POPN) | | 1.00 | –0.02 | 0.09 | –0.03 | 0.45[d] | 0.05 |
| Altitude (ALT) | | | 1.00 | –0.15 | 0.30[c] | 0.12 | –0.22[b] |
| Rainfall (RAIN) | | | | 1.00 | 0.45[d] | 0.17 | 0.49[d] |
| Volcanic ferrisols (SOIL)[d] | | | | | 1.00 | –0.14 | 0.37[d] |
| Remoteness (REM) | | | | | | 1.00 | 0.25[b] |
| Latitude (LAT) | | | | | | | 1.00 |

[b]$P < 0.05$. [c]$P < 0.01$. [d]$P < 0.001$. Estimated near each centre as the approximate percentage area of ferrisols (mostly on basic volcanic rocks) plus one-quarter of the approximate percentage of associated ferralitic soils (mostly on non-volcanic rocks and often occurring widely in ferrisol-rich regions).

### Estimation of incidence rates and adjustment for underreporting

Given the scarcity of medical facilities in Africa, many areas are not within easy reach of a hospital and so, except for a few urban centres, a general underreporting of cancer is inevitable. Knowledge of the relative stability of total (compared with individual) cancer rates around the world, especially within continents (Doll et al, 1966; 1970), together with indications of much sharper gradients of frequency for individual sites in Africa than are common in the West (Cook and Burkitt, 1971), has been used to establish a standard against which to adjust the observed incidence rates for individual types of cancer. The basis of adjustment for underreporting has been to assume that the level of incidence everywhere in Africa was similar, for a core group of malignancies, to the average rate for these pooled cancers in four centres with particularly high standards of cancer registration that had previously published incidence rates: Johannesburg and Natal in South Africa (Higginson and Oettle, 1960; Schonland and Bradshaw, 1968); Ibadan, Nigeria (Edington, 1970); and Kampala, Uganda (Davies et al, 1962), though using the 1964–68 data for Kampala rather than the earlier published series. The average rates for the four centres used as standard are referred to as the '4-registry' rates.

The core group of malignancies for which the assumption of equal incidence has been made consists of all malignancies other than those that showed considerable geographical variation within Africa (from common or relatively common to almost non-existent within short distances); the latter comprise cancers of the oesophagus, lung, penis, bladder, nasopharynx and BL. Cancers of the cervix and breast have also been omitted from the total because, although they show less marked geographical variation and there are no areas where either is very rare, whichever of the two predominates locally tends to be by far the most common cancer diagnosed in women. Relatively slight variations in incidence would, therefore, be reflected in the pooled incidence of the core malignancies. The core group of malignancies derived by excluding these sites is referred to subsequently as the 'less variable' (LV) tumours.

Age-standardized incidence ratios (SIRs) were calculated for each local area using expected values derived from application of standard age-specific rates to local populations. Relatively good census data, with an age breakdown, became available during the course of the East and Central African cancer surveys for most of the territories involved and for these it has been possible to use local population data. Overall, the data suggest a broad similarity of age structure common to rural areas in Africa and another pattern for cities. In the latter there is an excess of young adults, particularly of young men, who have come to town to seek work. With this knowledge, when detailed local population data were not available for areas outside East and Central Africa, estimates of age structure were taken either from average 'urban', 'rural' or combined 'urban/rural' population structures, as appropriate, or from similar neighbouring areas. The method used for adjusting for underreporting requires an estimation only of the age structure of populations not their total size.

The SIRs for individual malignancies were multiplied by the 'standard' cumulative rates for each type of cancer (age 0–64 years) (Day, 1976) to convert them to rates. The age group 0–64 years was chosen in view of the young age structure of African populations and of the underuse of medical facilities by elderly patients. The expected and observed values used were for persons of all ages, but the majority of patients were below age 65 in series

© Cancer Research Campaign 1998

**Table 3** Relative risks of Kaposi's sarcoma and lymphomas associated with different geographical variables in sub-Saharan Africa

| | **Relative risks** | | | | ***P*-values for** | |
|---|---|---|---|---|---|---|
| | | | | | **Heterogeneity** | **Trend** |
| *Moisture index* | **Dry** | **Moderately dry** | **Moderately humid** | **Humid** | | |
| Kaposi's sarcoma | 1.00 | 1.69 | 1.85 | 2.33 | 0.02 | 0.007 |
| Burkitt's lymphoma | 1.00 | 3.47 | 1.45 | 1.34 | 0.005 | 0.05 |
| Non-Burkitt's lymphoma | 1.00 | 1.48 | 1.25 | 1.59 | 0.07 | 0.12 |
| NHL | 1.00 | 1.34 | 1.21 | 1.67 | 0.042 | 0.02 |
| Hodgkin's lymphoma | 1.00 | 1.91 | 1.39 | 1.31 | 0.31 | 0.79 |
| *Population density* | Low | Moderate | High | Urban | | |
| Kaposi's sarcoma | 1.00 | 1.37 | 1.4 | 0.37 | < 0.001 | 0.005 |
| Burkitt's lymphoma | 1.00 | 1.74 | 1.17 | 0.95 | 0.27 | 0.68 |
| Non-Burkitt's lymphoma | 1.00 | 1.14 | 1.23 | 1.09 | 0.68 | 0.51 |
| NHL | 1.00 | 1.09 | 1.15 | 1.05 | 0.87 | 0.7 |
| Hodgkin's lymphoma | 1.00 | 1.24 | 1.46 | 1.2 | 0.77 | 0.5 |
| *Altitude* (in feet) | < 2000 | 2000– | 4000– | 5000+ | | |
| Kaposi's sarcoma | 1.00 | 2.94 | 2.22 | 2.53 | <0.001 | 0.003 |
| Burkitt's lymphoma | 1.00 | 0.95 | 1.02 | 0.19 | < 0.001 | 0.04 |
| Non-Burkitt's lymphoma | 1.00 | 1.03 | 1.03 | 0.82 | 0.57 | 0.42 |
| NHL | 1.00 | 1.1 | 1 | 0.78 | 0.32 | 0.25 |
| Hodgkin's lymphoma | 1.00 | 0.79 | 1.04 | 0.83 | 0.79 | 0.86 |
| *Rainfall* (in mm) | < 1000 | 1000– | 1400– | 1800+ | | |
| Kaposi's sarcoma | 1.00 | 1.62 | 2.17 | 1.51 | 0.007 | 0.02 |
| Burkitt's lymphoma | 1.00 | 2.31 | 1.51 | 1.68 | 0.14 | 0.58 |
| Non-Burkitt's lymphoma | 1.00 | 1.66 | 1.61 | 1.8 | < 0.001 | 0.003 |
| NHL | 1.00 | 1.55 | 1.77 | 1.97 | < 0.001 | < 0.001 |
| Hodgkin's lymphoma | 1.00 | 2.07 | 1.22 | 1.47 | 0.04 | 0.76 |
| *Volcanic ferrisols* (% area) | < 15% | 15%– | 35%– | 55%+ | | |
| Kaposi's sarcoma | 1.00 | 1.8 | 2.23 | 3.11 | < 0.001 | < 0.001 |
| Burkitt's lymphoma | 1.00 | 1.04 | 0.99 | 0.48 | 0.35 | 0.16 |
| non-Burkitt's lymphoma | 1.00 | 1.11 | 1.15 | 1.15 | 0.85 | 0.44 |
| NHL | 1.00 | 0.98 | 1.07 | 1.17 | 0.7 | 0.27 |
| Hodgkin's lymphoma | 1.00 | 1.73 | 1.53 | 1.12 | 0.19 | 0.85 |
| *Remoteness/westernization* (Wn) | Remote | Moderate | Agriculture Wn | Tourist/industrial Wn | | |
| Kaposi's sarcoma | 1.00 | 0.84 | 0.62 | 0.34 | < 0.001 | < 0.001 |
| Burkitt's lymphoma | 1.00 | 0.64 | 0.52 | 0.59 | 0.37 | 0.14 |
| non-Burkitt's lymphoma | 1.00 | 0.87 | 0.89 | 0.8 | 0.7 | 0.3 |
| Lymphosarcoma | 1.00 | 0.81 | 0.79 | 0.77 | 0.42 | 0.14 |
| Hodgkin's lymphoma | 1.00 | 1.13 | 1.3 | 0.95 | 0.81 | 0.8 |
| *Latitude* | 0–5°N/S | 5–15°S | 15–25°S | 25°S | | |
| Kaposi's sarcoma | 1.00 | 0.56 | 0.44 | 0.31 | < 0.001 | < 0.001 |
| Burkitt's lymphoma | 1.00 | 1.01 | 0.32 | 0.22 | < 0.001 | < 0.001 |
| Non-Burkitt's lymphomas | 1.00 | 1.02 | 0.69 | 0.32 | < 0.001 | < 0.001 |
| NHL | 1.00 | 0.99 | 0.69 | 0.28 | < 0.001 | < 0.001 |
| Hodgkin's lymphoma | 1.00 | 1.19 | 0.7 | 0.47 | 0.06 | 0.08 |
| *Total observed tumours* | | | | | | |
| Kaposi's sarcoma | 1074 | Non-Burkitt's lymphomas | 2273 | | | |
| Burkitt's lymphoma | 955 | NHL | 690 | | | |
| | | Hodgkin's lymphoma | 583 | | | |

[a]The column headings used for the moisture index are abbreviations for combinations of mapped categories (Grove et al, 1967) as follows: 'dry' – 'arid', 'semiarid' and 'semiarid/dry subhumid'; 'moderately dry' – 'dry subhumid' and 'dry subhumid/moist subhumid'; 'moderately humid' – 'moist subhumid' and 'moist subhumid/humid'; 'humid' – 'humid' and 'very humid'.

where the age of patients was known and so linking the SIRs to 0–64 cumulative rates gives the most appropriate comparison with rates from outside Africa. For malignancies other than those of particular interest in the present paper, expected values were derived using the '4-registry' age-specific rates. BL was not classified separately from other NHL in the Ibadan data, or KS from other connective tissue tumours in any of the centres except Kampala. Average East African age-specific rates (adjusted for underreporting by reference to the '4-registry' LV rates) derived from the MRC frequency study were, therefore, used as standard for KS and BL, and average '4-registry' rates excluding Ibadan were used as standard for other NHL. The conversion of the SIRs to estimated rates centred on the 'standard' cumulative rate means that the use of different 'standards' for different malignancies will have little effect on the final estimates. The adjustment for underreporting of the estimated rates was made by multiplying the local rates for individual sites by the '4-registry' rate for the LV tumours divided by the local LV tumours rate.

© Cancer Research Campaign 1998

A few of the published frequency series that have been incorporated in the study comprised only malignancies diagnosed at a central histopathology laboratory and occasionally, in regional breakdowns of the data, only details of selected sites. For these series, knowledge gained from the experience of cancer registration within East and Central Africa, with regard to the frequency of referral to central hospitals for different sites of malignancy and the frequency with which biopsies are taken from different sites, was used to provide additional adjustment to allow for site-specific underreporting by changing the balance of accessible and inaccessible tumours in the total.

Incomplete regional data have been used in the present study, for example from Cameroon and Zaire, only where numbers have been given both for the specific tumours (KS) and for the total number of all malignancies. In these instances, a profile of 'less variable' tumours was built up from knowledge of the relative frequency of individual sites in national data or in neighbouring territories. In a few instances, the number of KS or BL tumours, categorized as connective tissue or NHL in the published series, was estimated from comments in the literature. In adjusting for underreporting, an exceptionally high frequency at a site included in the LV tumours was adjusted downward to an average level, as, for example, with liver cancer in Maputo, which would otherwise have accounted for two-thirds of the LV total. (Details of all local adjustments are given in the notes accompanying Appendix Table 1 while the rates appear on the maps with a qualifying 'e'.)

Sensitivity analyses were carried out to establish the likely limits of error inherent in the estimations of incidence, given that the rates for the LV tumours may themselves have been subject to some underreporting or been less homogeneous than anticipated. Estimates of incidence were recalculated with the LV age-specific and cumulative rates (used as standard) set equal to the highest of the '4-registry' rates, those observed in Natal, rather than to the average values, and, alternatively, to the rates for the same period in a black population living outside Africa, in Jamaica (Brooks, 1976). In addition the effect of excluding KS and NHL themselves from the LV tumours was also considered.

### Mapping of estimated incidence rates

Maps were prepared in which estimated cumulative rates (per 1000 population at risk) were plotted on the local areas to which they refer. The male rates for KS, BL and NHL and the female rate for KS are shown in Figures 1. Correlation coefficients for the association between male and female rates for the different lymphomas show a similar pattern of occurrence (Table 1).

### Associations of incidence with geographical parameters

For sub-Saharan Africa, relative risks associated with a number of geographical variables were derived by logistic regression so as to characterize the areas of high and low incidence for the malignancies of interest. The variables [moisture index, a combination of rainfall and evaporation (Grove, 1967); population density; altitude; rainfall; occurrence of ferrisols, a soil type that has been suggested as a risk factor for KS (Zieglar, 1994); remoteness, i.e. from industrialization and westernization; and latitude] were either read from maps or derived from geographical knowledge of the different areas during the relevant time period (Lewis et al, 1967; Harrison Church et al, 1967).

The logistic regressions (Glim, 1987) were carried out using the summed male and female observed values for the malignancies of interest as the dependent variable and offsetting the logarithm of the expected values (adjusted for underreporting). Use of the pooled male and female data allowed a finer areal subdivision in some regions than was used for the purposes of mapping, when sex-specific rates were used so as to permit international comparisons. There is a considerable degree of correlation between the environmental and social variables included in the logistic regression analyses (coefficients are given in Table 2). However, in view of the approximate nature of such geographical comparisons, lacking as they do information on the exposure levels of individual patients, no attempt was made to apportion risk more specifically in multifactorial analyses.

### RESULTS AND DISCUSSION

### Kaposi's sarcoma

The highest rates of more than 6 per 1000 were found in a broad strip across equatorial Africa, with particularly high rates in north-eastern Zaire and in western Uganda and Tanzania (Figure 1A). In addition, narrow belts of high incidence stretched westward from eastern Zaire to the coast in Cameroon and southward down the Rift Valley into Malawi. Assumption of a level of incidence for the LV tumours as high as the level of incidence in Natal, or exclusion of KS and NHL from the LV tumours, raises the incidence of the highest estimated rates from 1 to 2 per 1000. Setting the level of the LV tumours equal to the rates observed in Jamaica gives increases up to 3 per 1000, giving a probable maximum cumulative incidence of c. 15 per 1000. By comparison, calculation of cumulative rates from European data (Cottoni et al, 1996; Grulich et al, 1992), suggest a highest male incidence of 0.8 per 1000 for Sardinia and a rate of only 0.05 per 1000 for England and Wales. The estimated cumulative rates for KS in western Uganda and eastern Zaire were on a par with those for cancer of the colon in much of Western Europe (one of the commoner Western malignancies) (Muir et al, 1987).

KS in Africa was about eight to ten times more common in men than in women. The rates for the two sexes were less closely correlated than the rates for the lymphomas (Table 1). However, the broad zone within which rates were elevated above those observed elsewhere in the world was similar to that for males (Figure 1B).

The relative risks associated with the geographic variables (Table 3) characterize the areas within which KS was common, with the highest levels near the equator; in areas with an altitude over 2000 ft; in areas with moderate to high humidity; in areas that are mostly rural and remote from westernization (although densely populated); and in areas that are likely to have ferrisol and ferralitic soils. Significant trends were observed for all these variables. The conditions outlined are typical of the high-incidence areas in the volcanic mountains that fringe the western branch of the great Rift Valley in eastern Zaire, in western Uganda and Tanzania, and further south in Malawi. Volcanic mountains also occur on the west of the continent in the mountainous areas of Cameroon where the incidence again appears to be elevated. However, there is some indication that, in this respect, there were anomalously low rates in Rwanda and Burundi on the watershed between Zaire and Uganda and Tanzania (Figure 1A and Appendix Table 1). In central Kenya, the physical conditions are similar to those of the Rift Valley in Uganda and Zaire, although

© Cancer Research Campaign 1998

**Figure 1** Estimated cumulative incidence rates per 1000 (age 0–64). (**A**) Kaposi's sarcoma, males. (**B**) Kaposi's sarcoma, females. (**C**) Burkitt's lymphoma, males. (**D**) Non-Hodgkin's, non-Burkitt's lymphoma, males. $2_e$ = centres where the estimates of cumulative incidence rest on specific assumptions, outlined in the text, and described in full in the notes accompanying Appendix Table 1. Centres where a zero rate is based on no cases or represents a rate of < 0.05 are shown as 'o' instead of '0' in **B**. Rates from series with LV tumours < 100 are shown in smaller typeface the incidence is only moderate. However, in the extensive volcanic mountains of Ethiopia, which are as remote as those of eastern Zaire and south-west Uganda, are almost as densely populated and have widespread areas of ferrisols, there were indications of only a low incidence of KS (Burkitt, 1966; Lester and Tsega, 1976).

The broad range of altitude, and thus temperatures, at which the incidence is elevated, and the broad range of moisture zones (in contrast to the pattern for BL), do not indicate clear climatic limits on the occurrence of the disease in agreement with a previous study (Taylor et al, 1972). A recent study from Italy found a higher risk for classic (pre-AIDS) KS in persons born in areas where malaria is endemic (Geddes et al, 1995). In Africa, however, many of the highest estimated rates occur in areas of high altitude where malaria is almost unknown.

© *Cancer Research Campaign 1998*

Recent publications of cancer incidence in Africa suggest dramatic increases in the incidence of KS over the last 10–15 years (Wabinga et al, 1993; Basset et al, 1995; Newton et al, 1996), presumably as a result of the HIV epidemic. Adjustment for under-reporting among the recent data for males from Butare, Kampala and Harare suggests incidence rates that by 1990 had almost doubled in Rwanda, to over 5 per 1000, and had increased around 20-fold in Uganda and Zimbabwe, to over 55 per 1000 in Kampala and to around 22 per 1000 in Harare (Appendix Table 1). The rather modest rate in Rwanda following the start of the HIV epidemic is consistent with the low rates in the 1960s described above for Rwanda and Burundi and also with the relatively low seroprevalence rates of HIV reported from much of Rwanda, around 10% in towns such as Butare (Rwanda HIV Study Group, 1989) compared with 40% in parts of Kampala (Ugandan Ministry of Health, 1997).

The recently discovered HHV-8 is now considered by many to correlate geographically in its occurrence with the occurrence of KS and not to be generally ubiquitous in human populations (Gao et al, 1996). If this is true, then the pattern of incidence shown here (Figure 1A) may possibly mirror the distribution of the virus prior to the advent of AIDS. This suggests that there may have been a previous spread of infection from an original focus in high-altitude north-eastern Zaire, perhaps following routes of trade or of migration in search of work. Temporary migration of workers from Zambia and Malawi both to the copper mines in Katanga, Zaire and to the gold mines in South Africa could explain the occurrence of KS as far south as the northern Transvaal and the south of Mozambique. Similarly, the annual pilgrimage to Mecca from the Muslim north of West Africa and even trade between the old inland kingdoms of West Africa and southern Europe may have contributed to the spread of the disease to Mediterranean countries, where it was more common than in non-Mediterranean Europe prior to the advent of AIDS (Qunibi et al, 1988; Geddes et al, 1994).

### Burkitt's lymphoma

The map of BL (Figure 1C) shows, in terms of incidence, the pattern of frequency that has previously been described in great detail for this malignancy (Burkitt, 1962*a*,*b*, 1963, 1964). The highest incidence rates are estimated at between 4 and 5 per 1000 in the West Nile district of Uganda and in the Mara, Mwanza and Shinyanga districts of Tanzania. Most cases occurred in those under the age of 20 and the rates compared with an incidence of only 0.8 per 1000 for leukaemia among young males in the UK (Waterhouse et al, 1976).

The estimated cumulative incidence of BL has increased in Kampala, Uganda, between 1964–68 and 1989–93, from 0.2 to 0.8 per 1000 for males and from 0.04 to 0.5 per 1000 for females (an average sevenfold increase for both sexes combined, $P < 0.001$). All the recent cases occurred in children (Wabinga et al, 1993), and there is thus no sign of an increase in young adults such as has occurred in the West in association with HIV. However, it is not known whether the children with BL were HIV positive. There is no sign of an increase in BL at the other centres, Butare and Harare, for which recent data are available (Appendix Table 1).

The areas of particularly high incidence have been defined as areas where malaria is hyperendemic (Burkitt, 1969), and the present study gives no evidence that does not support the possible relationship with malaria. The declining relative risks with increasing westernization (Table 3) include areas such as Kinshasa and Kampala where effective malaria eradication was thought to have limited the disease despite conditions that were suited to its occurrence (Burkitt, 1969). The same explanation has been also given for the very low frequency of the disease noted in the island of Zanzibar (Chopra, 1968). It is not known whether the increase in BL apparent in 1989–91 in Kampala follows an increase in the frequency of malaria.

### Non-Hodgkin's lymphoma, excluding Burkitt's lymphoma

Features of note in the map of NHL (Figure 1D) are a relatively low incidence throughout southern Africa with rates mostly of 1 per 1000; a broad belt of elevated incidence throughout tropical Africa (from Malawi, Zambia and Angola in the south to Senegal and the Sudan in the north) with rates in the range 2–4 per 1000 over most of central and East Africa; and (in contrast to the maps for KS and BL) relatively high rates of 4 and 5 per 1000 in North Africa. In the island of Madagascar the rate was similar to that of central rather than southern Africa. There were certain small centres in the tropical belt where very few lymphoid tumours were diagnosed, and these are of suspect validity.

The only geographical variables (apart from latitude) to show an association with the occurrence of NHL were rainfall and the related 'moisture index' (Table 3). The relative risks showed a clear trend of incidence from areas with an annual rainfall of less than 1000 mm to those with more than 1800 mm. There was no indication of a gradient with altitude that might suggest an effect of temperature or the influence of ultraviolet radiation (Adami et al, 1995).

Analysis of 1967–73 data from Uganda showed a geographical association for the occurrence of BL and other NHL (especially high-grade lymphomas) Schmauz et al, 1990). Calculation of correlation coefficients for the sub-Saharan areas shown on the maps (excluding the areas of suspiciously low lymphoma rates mentioned above) confirms this association over a much broader area ($r_S$ 0.455, $P < 0.001$). The coefficients for KS and BL or KS and other NHL were both non-significant (0.104 ($P > 1.0$) and 0.224 ($P = 0.1$).

Comparison of rates for NHL in Africa with rates typical for other continents during similar periods (using data from Doll et al, 1966, 1977; Waterhouse et al, 1976) shows a mid-range cumulative incidence (age 0–64 years) around Kampala and in Dakar (Senegal) very similar to that in Birmingham, UK. In contrast, the rates from southern Africa (for Bulawayo, Natal and Johannesburg) are very low – lower even than those reported from India and Japan. The rates for the black population of San Francisco were intermediate between the Senegal/Uganda rates and the high (pre-AIDS) rates for the white population of San Francisco. Assumption of a general cancer incidence in Africa as high as in Natal or Jamaica, or exclusion of KS and BL from the LV tumours, would raise the estimated rates for Kampala and Dakar to around 4 per 1000 and put them on a par with those for the black population of San Francisco.

The relatively high rates for other NHL in North Africa partly reflect the high incidence of intestinal lymphomas associated with malabsorption syndrome that occurs to the south of the Mediterranean and in the Middle East. In sub-Saharan Africa, study of 44 gastrointestinal lymphomas diagnosed in Uganda between 1964 and 1975 found no indication of associated malabsorption syndrome (Owor et al, 1977).

There has been no significant increase in the incidence of NHL at any of the three centres (Butare, Kampala and Harare) for which recent data are available.

It is possible that there has been some confusion in the diagnoses of Hodgkin's and NHL, particularly in areas where medical

 © Cancer Research Campaign 1998

facilities are scarce. Even so, the combination of the two diseases would made little difference to the pattern of distribution for NHL since the estimated cumulative incidence for Hodgkin's lymphoma is similar throughout Africa, at around 1 per 1000 (Appendix Table 1 and Appendix Figure 4).

### CONCLUSIONS

Data have been presented for three types of cancer (KS, BL and NHL) which, in the West, have a raised incidence in association with HIV infection. All three were known or thought to have a relatively high frequency in tropical Africa but, for the first time, rates have been estimated across the continent that permit comparison with incidence rates elsewhere in the world and which give a more accurate picture of former levels of incidence within Africa than has previously been available. The maps and the comparisons with geographical variables emphasize the completely different distribution of KS and BL within the zone where they are both common and highlight the exceptionally high incidence of KS and BL there (prior to the start of the HIV epidemic) compared with the rest of the world. Other NHL shows some degree of association with the distribution of BL but appears to have occurred less frequently in central Africa than in many Western countries, whereas the rates for southern Africa were low.

### ACKNOWLEDGEMENTS

Our very sincere thanks to the many doctors and other staff at the very large number of small medical centres throughout Africa who over the years helped to complete the cancer returns; also, to the pathologists, surgeons and epidemiologists who were in charge of the national cancer registries: to MSR Hutt, MC Pike, and AC Templeton in Uganda; to H Cameron, M Rogoff and S Chopra in Kenya; to G Slavin and C Anderson in Tanzania; to the late J Borgstein and to H Spencer in Malawi; to the late R Carruthers and SB Bhagwandeen in Lusaka, Zambia; to E Rea, NP Desai and J Fine in Kitwe, Zambia; and to AM El Hassan and ES Doaud in the Sudan; also to the cancer registrars who undertook the collection and recording of the data.

### REFERENCES

Adami J, Frisch M, Yuen J, Glimelius B and Melbye M (1995) Evidence of an association between non-Hodgkin's lymphoma and skin cancer. *BMJ* **310**: 1491–1495

Bassett MT, Chikunongo E, Mauchaza B, Levy L, Ferlay J and Parkin DM (1995) Cancer in the African population of Harare, Zimbabwe in 1990–92. *Int J Cancer* **63**: 29–36

Beral V (1991) The epidemiology of cancer in AIDS patients. *AIDS* **5** (suppl. 2): S99–103

Brooks SEH (1976) Jamaica, Kingston and St Andrew. In *Cancer Incidence in Five Continents*, Vol. III, Waterhouse JAH, Muir C, Correa P and Powell J (eds), pp. 172–175. IARC: Lyon

Burkitt D (1962*a*) Determining the climatic limitations of a children's cancer common in Africa. *BMJ* **ii**: 1019–1023

Burkitt D (1962*b*) Lymphoma syndrome in African children. *Ann Roy Coll Surg* **30**: 211–219

Burkitt (1963) A children's cancer dependent on environment. In *Viruses, Nucleic Acids and Cancer*. Paper presented at The 17th Annual Symposium on Fundamental Cancer Research, 1963, pp. 615–629. Wilkins and Wilkins: Baltimore

Burkitt D (1964) A lymphoma syndrome dependent on environment. ii. epidemiological features. In *Symposium on Lymph Tumours in Africa*, Paris, 1963. pp.119–136. S Karger: Basle

Burkitt D (1966). Surgical pathology in the course of the Nile. *Ann Roy Coll Surgeons* **39**: 236–247

Burkitt DP (1969) Etiology of Burkitt's lymphoma – an alternative hypothesis to a vectored virus. *J Natl Cancer Inst* **42**: 19–28

Burkitt DP, Hutt MSR and Slavin G (1968) Clinico-pathological studies of cancer distribution in Africa. *Br J Cancer* **22**: 1–6

Chang Y, Cesarman E, Pessin MS, Lee F, Culpepper J and Moore PS (1994) Identification of Herpes-like DNA sequences in AIDS-associated Kaposi's sarcoma. *Science* **266**: 1865–1869

Chopra SA (1968) A nine year study of malignancy in the people of Zanzibar and Pemba. In *Cancer in Africa*, Clifford P, Linsell CA and Timms GL (eds). East African Publishing House: Nairobi

Cook PJ and Burkitt DP (1971) Cancer in Africa. *Br Med Bull* **27**: 14–20

Cottoni F, De Marco R and Montesu MA (1996) Classic Kaposi's sarcoma in North-East Sardinia. *Br J Cancer* **72**: 1132–1133

Davies JNP, Wilson BA and Knowelden J (1962) Cancer incidence of the African population of Kyadondo (Uganda). *Lancet* **ii**: 328–330

Day NE (1976) A new measure of age-standardised incidence, the cumulative rate. In *Cancer Incidence in Five Continents*, Vol. III, Waterhouse JAH, Muir C, Correa P and Powell J (eds) pp. 443–445. IARC: Lyon

Doll R, Payne P and Waterhouse J (1966) *Cancer Incidence in Five Continents*, Vol. I. Springer: Berlin

Doll R, Muir C and Waterhouse J (1970) *Cancer Incidence in Five Continents*, Vol. II. Springer: Berlin

Edington GM (1970) Nigeria, Ibadan. In *Cancer Incidence in Five Continents*, Vol. II, Doll R, Muir C and Waterhouse J (eds), pp. 90–93. Springer: Berlin

Gao S-J, Kingsley L, Li M, Zheng W, Parravicini C, Zeiglar JL, Newton R, Rinaldo CR, Saah A, Phair J, Detels R, Chang Y and Moore PS (1996). Kaposi's sarcoma-associated herpesvirus antibodies among Americans, Italians and Ugandans with and without Kaposi's sarcoma

Geddes M, Franceschi S, Barchielli A, Falcini F, Carli S, Cocconi G, Conti R, Crosignani P, Gafa L, Giarelli L, Vercelli M and Zanetti R (1994) Kaposi's sarcoma in Italy before and after the AIDS epidemic. *Br J Cancer* **69**: 333–336

Geddes M, Franceschi S, Balzi D, Arniani S, Gafa L and Zanetti R. (1995) Birthplace and classic Kaposi's sarcoma in Italy. *J Natl Cancer Inst* **87**: 1015–1017

GLIM (1987) *The Generalised Linear Interactive Modelling system; release 3.77.* National Algorithms Group: Oxford

Grove AT (1967) *Africa South of the Sahara.* OUP: Oxford

Grulich AE, Beral V and Swerdlow AJ (1992) Kaposi's sarcoma in England and Wales before the AIDS epidemic. *Br J Cancer* **66**: 1135–1137

Harrison-Church RJ, Clarke JI, Clarke PJ and Hendersen HJ (1967) *Africa and the Islands*. Longman: London

Higginson J and Oettle AG (1960) Cancer incidence in the Bantu and 'Cape Coloured' races of South Africa: Report of a cancer survey in the Transvaal (1953–55). *J Natl Cancer Inst* **24**: 589–671

Hutt MSR (1984) Kaposi's sarcoma. *Br Med Bull* **40**: 355–358

Lester FT and Tsega E (1976) The pattern of adult medical admissions in Addis Ababa, Ethiopa. *E Afr Med J* **53**: 618–634

Lewis C, Campbell JD, Bickmore DP and Cook KF (1967) *The Oxford Atlas.* OUP: Oxford

Luxton JC, Thomas JA and Crawford DH (1991) Aetiology and pathogenesis of non-Hodgkin's lymphoma in AIDS. *Cancer Surveys* **10**: 103–119

Muir C, Waterhouse J, Mack T, Powell J and Whelan S (1987) *Cancer Incidence in Five Continents*, Vol. V. IARC: Lyon

Newton R, Ngilimana PJ, Grulich A, Beral V, Sindikubwabo B, Nganyira A and Parkin DM (1996) Cancer in Rwanda. *Int J Cancer* **66**: 75–81

Oettle G (1962) Geographical and racial differences in the frequency of Kaposi's sarcoma as evidence of environmental or genetic causes. In *Symposium on Kaposi's Sarcoma.* Ackerman LV and Murray JF (eds) *Acta Unio Int Contra Cancrum*, **18**: 330–363

Owor R (1977) Malignant lymphoma of the gastrointestinal tract in Uganda; a report of 40 cases. *E Afr Med J* **54**: 666–669

Parkin DM (1986) *Cancer Occurrence in Developing Countries.* IARC Scientific Publication No. 75. IARC: Lyon

Qunibi W, Akhtar M, Sheth K, Earl Ginn H, Al-Furayh O, DeVol EB and Taher S. (1988) Kaposi's sarcoma: the most common cancer after renal transplantation in Saudi Arabia. *Am J Med* **84**: 225–232

Rwanda HIV Seroprevalence Study Group (1989) Nationwide community based serological survey of HIV1 and other human retrovirus infections. *Lancet* **i**: 941–943

Schmauz R, Mugerwa JW and Wright DH (1990) The distribution of non-Burkitt, non-Hodgkin's lymphomas in Uganda in relation to malaria endemicity. *Int J Cancer* **45**: 650–653

© Cancer Research Campaign 1998

Schonland M and Bradshaw E (1968) Cancer in the Natal African and Indian 1964–66. *Int J Cancer* **3**: 304–316

Templeton AC (1973) Soft tissue tumours. In *Tumours in a Tropical Country: a Survey of Uganda 1964–68*, Templeton AC (ed), pp. 234–269, Springer-Verlag: New York

Taylor JF, Smith PG, Bull D and Pike MC (1972) Kaposi's sarcoma in Uganda. *Br J Cancer* **26**: 483–497

Thijs A (1957) L'angiosarcomatose de Kaposi au Congo belge et au Ruanda-Urundi. *Ann Soc Belge Med Trop* **37**: 295–308

Uganda, Ministry of Health (1997) *STD/AIDS Control Programme. HIV/AIDS Surveillance Report*. Entebbe, Uganda

Wabinga HR, Parkin DM, Wabwire-Mangen F and Mugerwa JW (1993) Cancer in Kampala, Uganda, in 1989–91: changes in incidence in the era of AIDS. *Int J Cancer* **54**: 26–36

Wasifi A, El Hassan A and Zak F (1967) Kaposi's sarcoma in the Sudan. *Sudan Med J* **5**: 213–222

Waterhouse JAH, Muir C, Correa P and Powell J (1976) *Cancer Incidence in Five Continents*, Vol. III. IARC: Lyon

Zieglar JL (1994) Endemic Kaposi's sarcoma in Africa and local volcanic soils. *Lancet* **342**: 1348–1351

© Cancer Research Campaign 1998